# Supplementary material for: Nematicidal Coumarins from Cnidium monnieri Fruits and Angelica dahurica Roots and Their Physiological Effect on Pine Wood Nematode (Bursaphelenchus xylophilus)
Source: Molecules. 2023 May 15;28(10):4109. doi: 10.3390/molecules28104109 (PMC10223656; doi:10.3390/molecules28104109)
Supplement: Supplementary file 1 [file molecules-28-04109-s001.zip › molecules-2369821-supplementary.pdf]

Supplementary Materials

Nematicidal Coumarins from *Cnidium monnieri* fruits and *Angelica dahurica* roots and their physiological effect on pine wood nematode (*Bursaphelenchus xylophilus*)

NMR spectra (Figure S1-S8) of the isolated nemeticidal coumarins (Compounds **1-8**) in this research were listed as follows.

Compound **1**: EIMS m/z: 244.2 $[M]^+$. ^1H NMR (600 MHz, $CDCl_3$): δ7.60 (1 H, d, J=9.42 Hz, H-4), δ7.27 (1H, d, J=8.58 Hz, H-5), δ6.81 (1H, d, J=8.58 Hz, H-6), δ6.23 (1H, d, J=9.42 Hz, H-3), δ5.21 (1H, m, H-2’), 3.53 (2H, d, J=7.32 Hz, H-1’), δ1.83 (3H, s, H-4’), δ1.65 (3H, s, H-5’); ^{13}C NMR ($CDCl_3$): δ161.47 (C-2), δ160.32 (C-7), δ152.94 (C-9), δ143.81 (C-4), δ132.74 (C-3’), δ126.26 (C-5), δ121.21 (C-2’), δ119.87 (C-8), δ114.81 (C-10), δ113.13(C-3), δ107.43 (C-6), δ56.14 (OCH_3), δ25.89 (C-4’), δ22.02 (C-1’), δ18.01 (C-5’).

Compound **2**: EIMS m/z: 216.1 $[M]^+$. ^1H NMR (600 MHz, $CDCl_3$): δ8.11 (1H, d, J=9.78 Hz, H-4), δ8.09 (1H, d, J=2.22 Hz, H-2’), 7.65 (1H, s, H-5), 7.07 (1H, J=2.22 Hz, H-3’), 6.40 (1H, d, J=9.78, H-3), 4.14 (3H, s, OCH_3); ^{13}C NMR ($CDCl_3$): δ160.29 (C-2), δ148.41 (C-7), δ147.50 (C-2’), δ145.82 (C-4), δ142.99 (C-9), δ132.42 (C-8), δ126.47 (C-6), δ116.95 (C-10), δ114.48 (C-3), δ107.62 (C-3’), δ61.54 (8-OCH_3).

Compound **3**: EIMS m/z: 386.2 $[M]^+$. ^1H NMR (600 MHz, $CDCl_3$): δ7.61 (1H, d, J=9.48 Hz, 4-H), δ7.41 (1H, d, J=8.46 Hz, 5-H), δ7.08 (1H, q, J=7.08 Hz, -CH=C-CO), δ6.81 (1H, d, J=8.46 Hz, 6-H), δ6.21 (1H, d, J=9.48 Hz, 3-H), δ5.28 (1H, d, J=6.96 Hz, 3’-H), δ4.28 (1H, d, J=6.96 Hz, 2’-H), δ2.0 (3H, s, CH_3-C=O), δ1.71 (3H each, d, J=7.08 Hz, CH_3-CH=C), 1.58 (3H, s, CH_3-C=CH), δ1.24, 1.18 (3H each, s, CH_3-C-O).

Compound **4**: EIMS m/z: 246.1 $[M]^+$. ^1H NMR (600 MHz, $CDCl_3$): δ8.11 (1H, d, J=9.78 Hz, H-4), δ7.61 (1H, d, J=2.34 Hz, H-2’), δ6.99 (1H, d, J=2.34 Hz, H-3’), δ6.28 (1H, d, J=9.78 Hz, H-3), δ4.16, 4.15 (2H, s, 5, 8-OCH_3); ^{13}C NMR ($CDCl_3$): δ160.54 (C-2), δ150.12 (C-7), δ145.22 (C-2’), δ144.39 (C-9), δ143.82 (C-5), δ139.46 (C-4), δ128.36 (C-8), δ114.94 (C-6), δ113.00 (C-3), δ107.78 (C-10), δ105.17 (C-3’), δ61.80 (8-OCH_3), δ60.94 (5-OCH_3).

Compound **5**: EIMS m/z: 246.1 $[M]^+$. 1H NMR (600 MHz, $CDCl_3$): δ7.62 (1H, d, J=9.84 Hz, H-4), 7.34 (1H, s, H-5), 6.81 (1H, s, H-8), 6.28 (1H, d, J=9.84 Hz, H-3), 5.37 (1H, t, J=8.52 Hz, H-2’), 3.93 (1H, m, H-3’), 1.34, 1.28 (3H each, s, 2CH_3).

Compound **6**: EIMS m/z: 270.1 $[M]^+$. ^1H NMR (600 MHz, $CDCl_3$): δ8.15 (1H, d, J=9.78 Hz, H-4), δ7.58 (1H, d, J=2.34 Hz, H-2’), δ7.15 (1H, s, H-8), δ6.94 (1H, d, J=2.34 Hz, 3’-H), δ6.26 (1H, d, J=9.78 Hz, 3-H), δ5.53 (1H, t, J=6.96 Hz, 3”-H), δ4.91 (2H, d, J=6.96 Hz, 2”-H), δ1.79, 1.69 (3H each, s, =C-$(CH_3)_2$); ^{13}C NMR ($CDCl_3$): δ161.31 (C-2), δ158.23 (C-7), δ152.78 (C-9), δ149.06 (C-5), δ144.98 (C-2’), δ139.92 (C-3’), δ139.64 (C-10), δ119.20 (C-6), δ114.33 (C-3), δ112.69 (C-4), δ107.65 (C-8), δ105.12 (C-3”), δ94.35 (C-4”), δ69.85 (C-2”), δ25.89, 18.30 (2CH_3).

Compound **7**: EIMS m/z: 270.1 $[M]^+$. ^1H NMR (600 MHz, $CDCl_3$): δ7.75 (1H, d, J=9.54 Hz, H-4), δ7.68 (1H, d, J=2.22 Hz, H-2’), δ7.34 (1H, s, H-5), δ6.80 (1H, d, J=2.22 Hz, H-3’), δ6.35 (1H, d, J=9.54 Hz, H-3), δ5.60

(1H, t, J=7.20 Hz, H-3”), δ5.00 (2H, d, J=7.20 Hz, H-2”), δ1.73, 1.71 (3H each, s, =C-$(CH_3)_2$); ^{13}C NMR ($CDCl_3$): δ160.60 (C-2), δ148.73 (C-7), δ146.70 (C-9), δ144.41 (C-5), δ143.94 (C-2’), δ139.83 (C-3’), δ131.79 (C-8), δ125.94 (C-6), δ119.87 (C-3), δ116.59 (C-4), δ114.81 (C-10), δ113.22 (C-3”), δ106.79 (C-4”), δ70.26 (C-2”), δ25.90, 18.20 ($2CH_3$).

Compound **8**: EIMS m/z: 216.1 $[M]^+$. ^{1}H NMR (600 MHz, $CDCl_3$): δ8.14 (1H, d, J=9.78 Hz, H-4), δ7.58 (1H, d, J=2.34 Hz, H-2’), δ7.12(1H, s, H-8), δ7.00 (1H, d, J=2.34 Hz, H-3’), δ6.26 (1H, d, J=9.78 Hz, H-3), δ4.26 (3H, s, 5-OCH_3); ^{13}C NMR ($CDCl_3$): δ161.29 (C-2), δ158.48 (C-7), δ152.83 (C-9), δ149.68 (C-5), δ144.88 (C-2’), δ139.32 (C-4), δ112.80 (C-6), δ112.68 (C-3), δ106.54 (C-10), δ105.11 (C-3’), δ93.97 (C-8), δ60.19 (5-OCH_3).

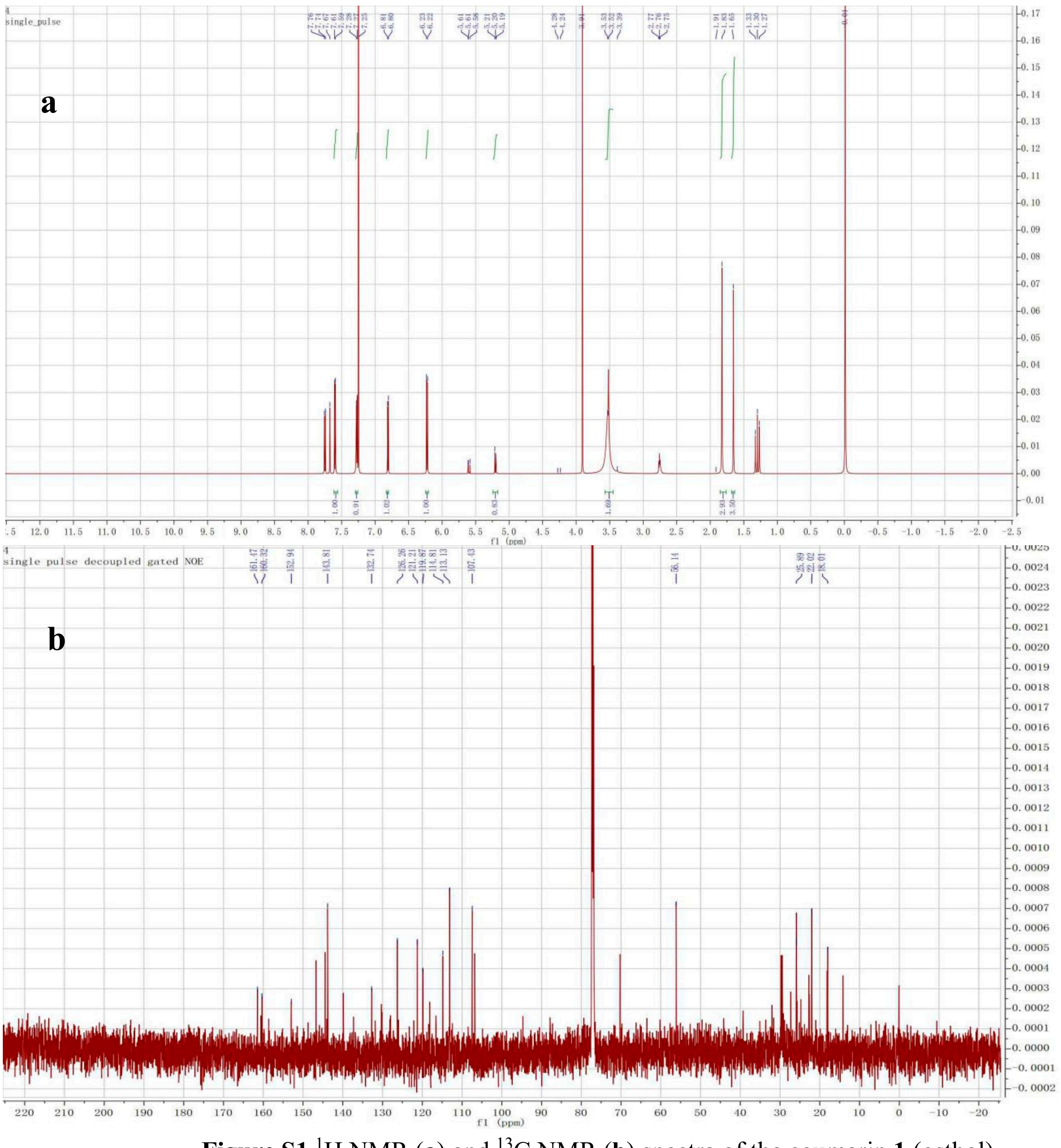

Figure S1 ^{1}H NMR (**a**) and ^{13}C NMR (**b**) spectra of the coumarin **1** (osthol)

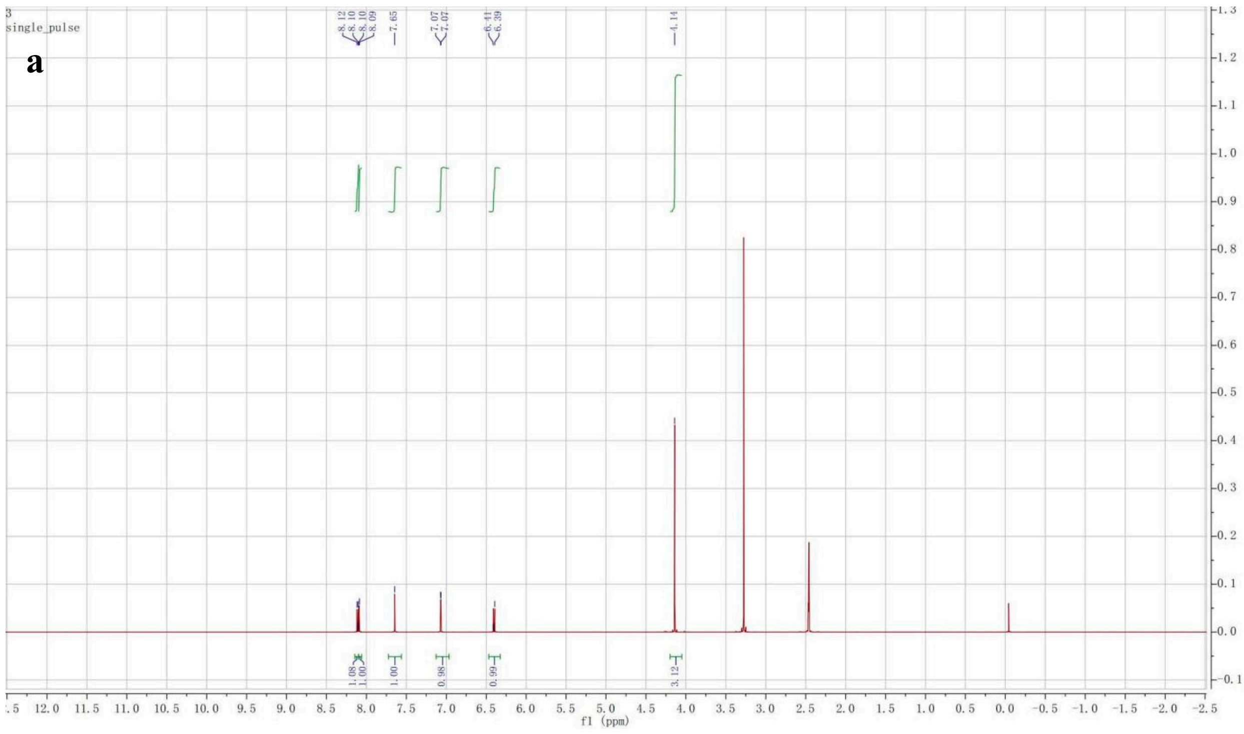

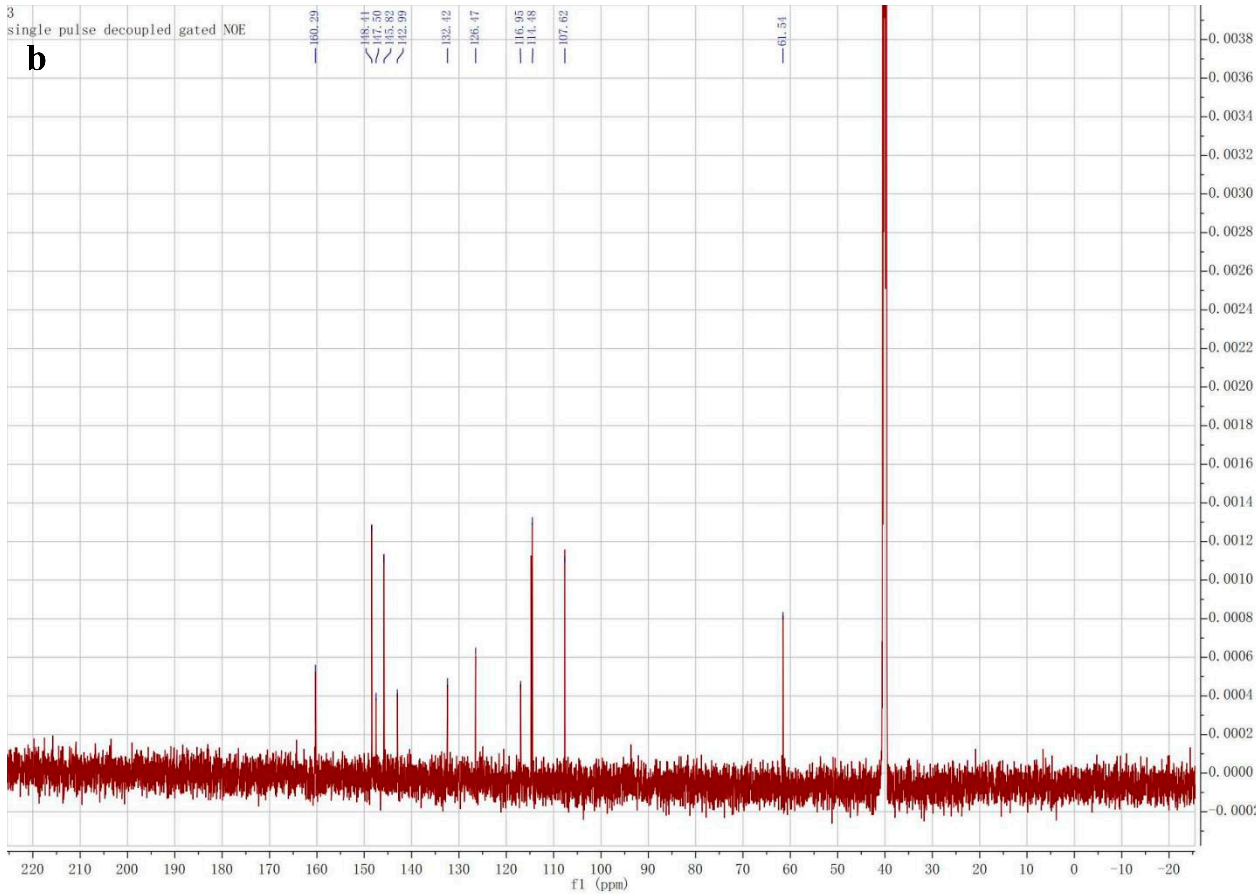

Figure S2 ^{1}H NMR (**a**) and ^{13}C NMR (**b**) spectra of the coumarin **2** (xanthotoxin)

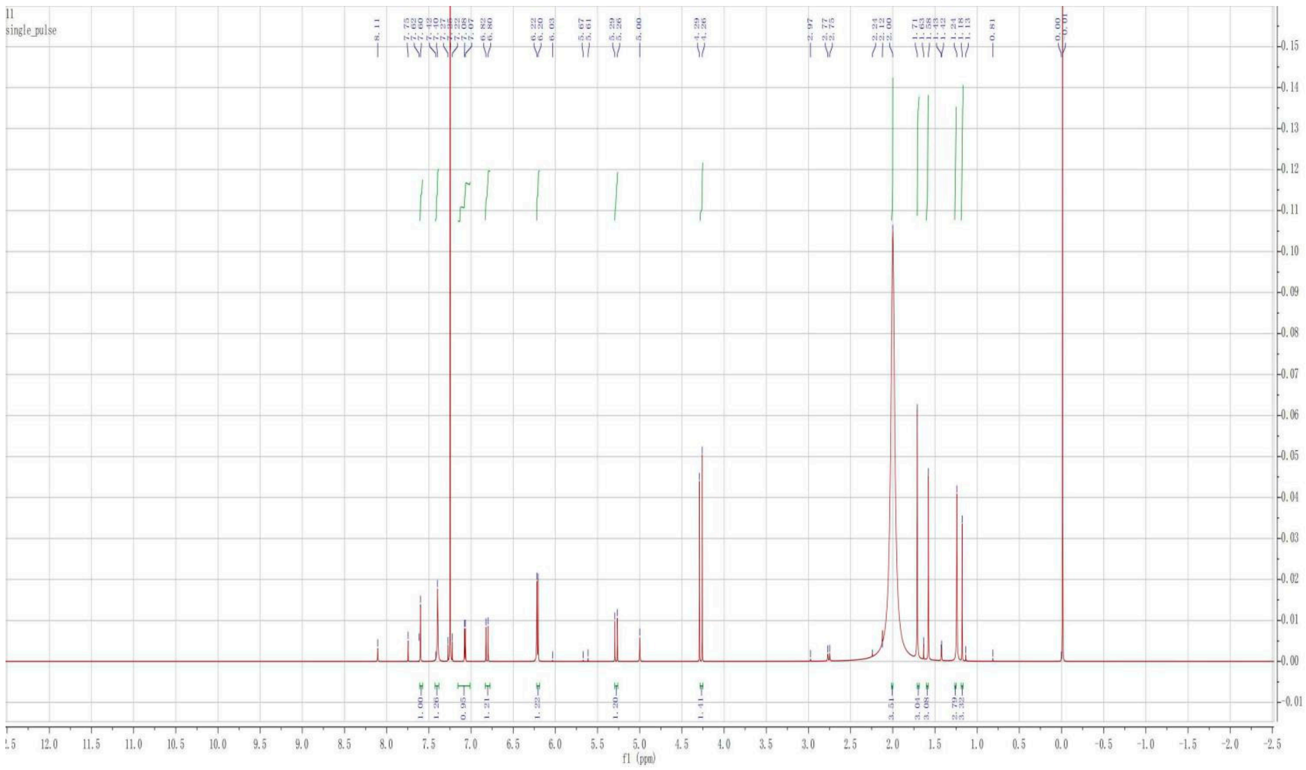

Figure S3 ^{1}H NMR spectrum of the coumarin **3** (cindimine)

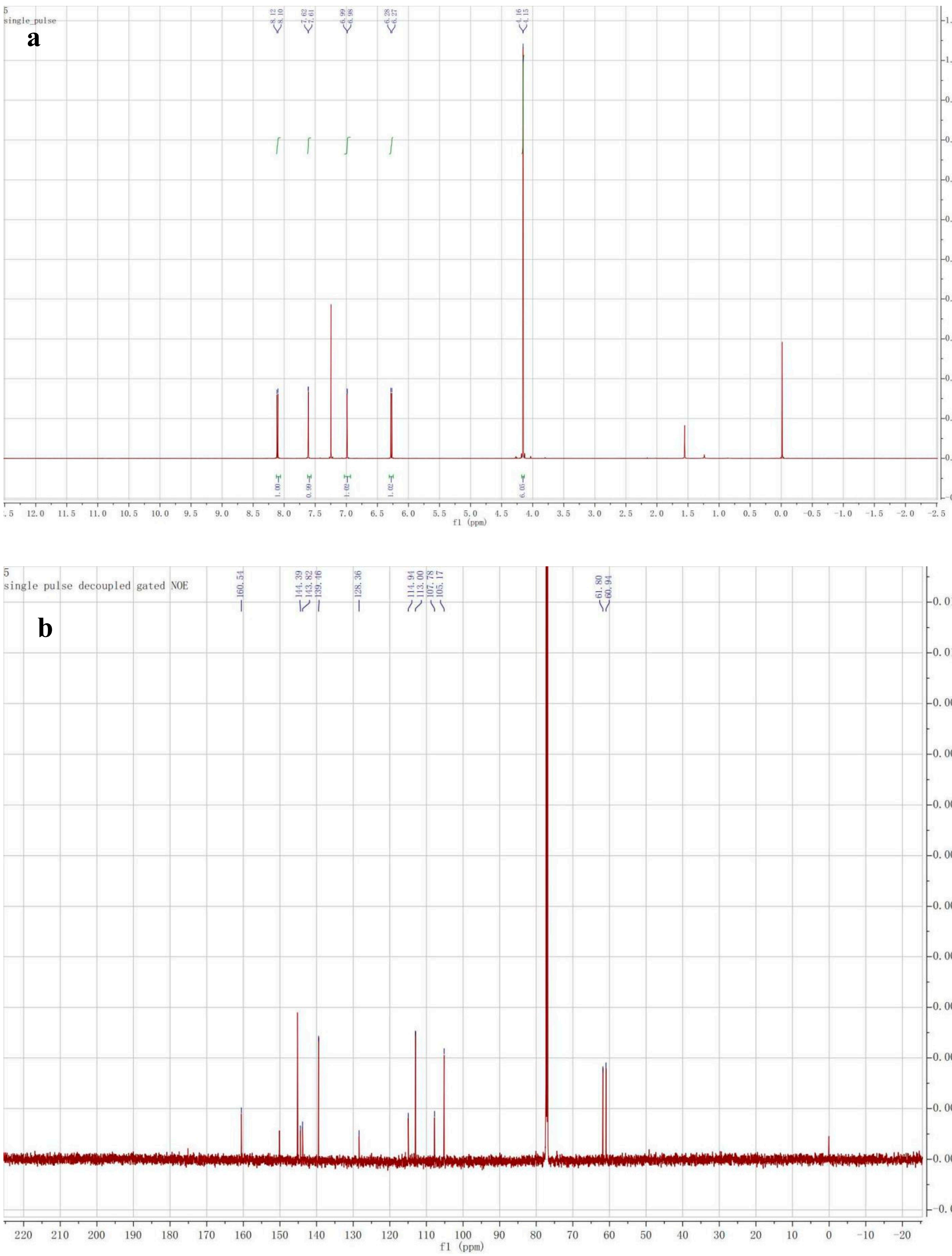

Figure S4 ^{1}H NMR (**a**) and ^{13}C NMR (**b**) spectra of the coumarin **4** (isopimpinellin)

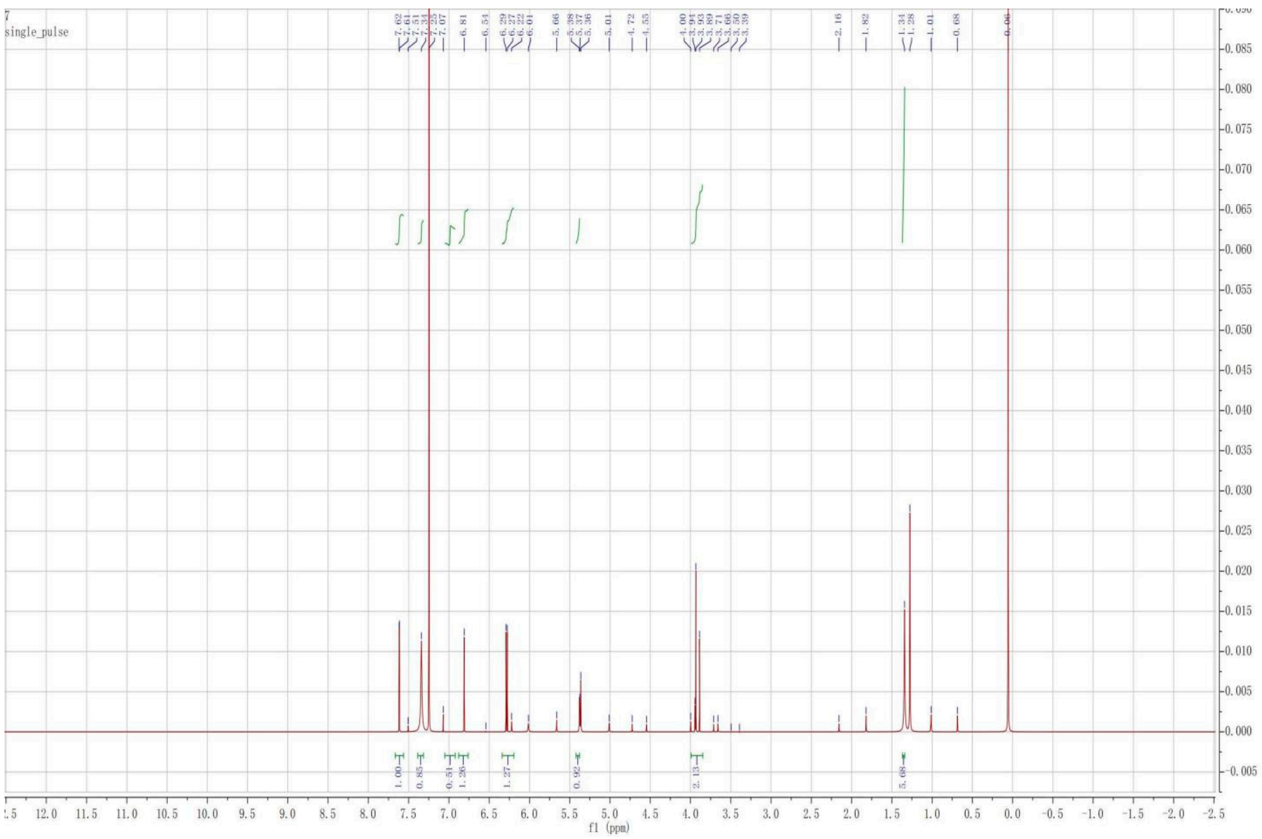

Figure S5 ^{1}H NMR spectrum of the coumarin **5** (marmesin)

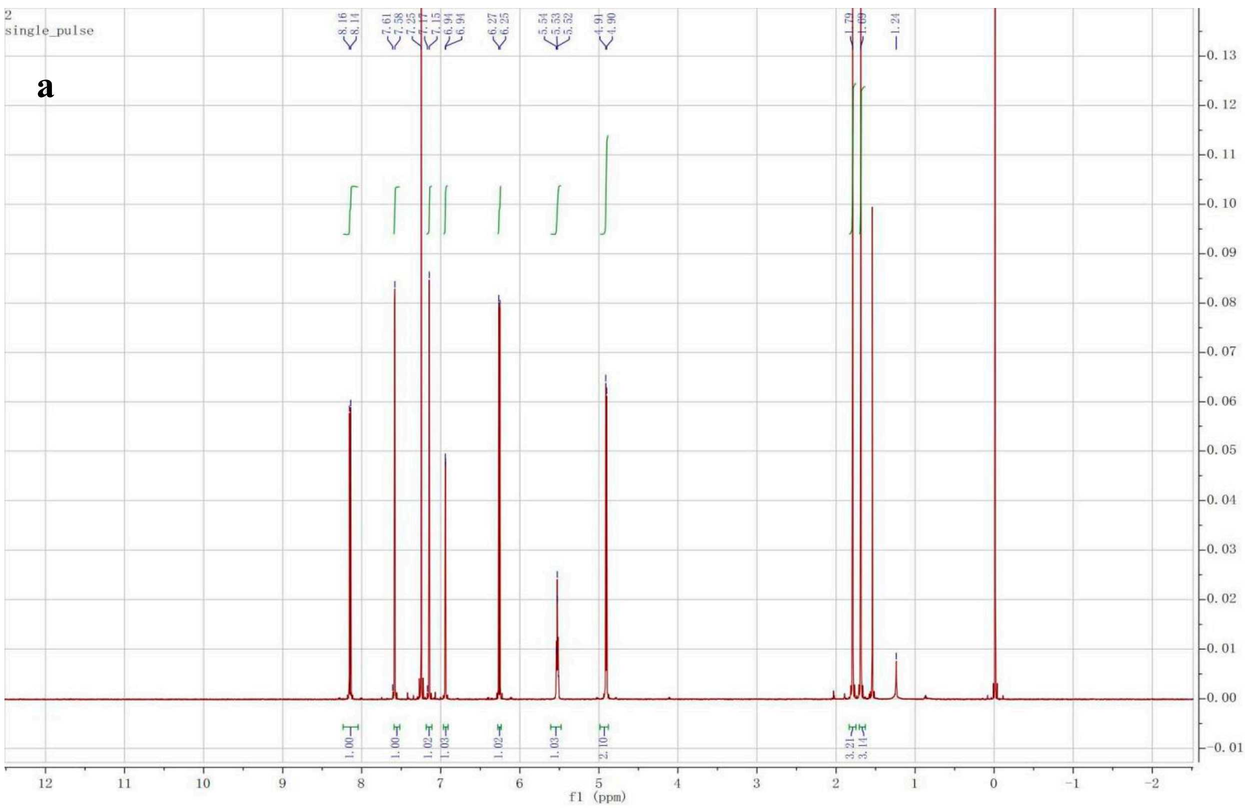

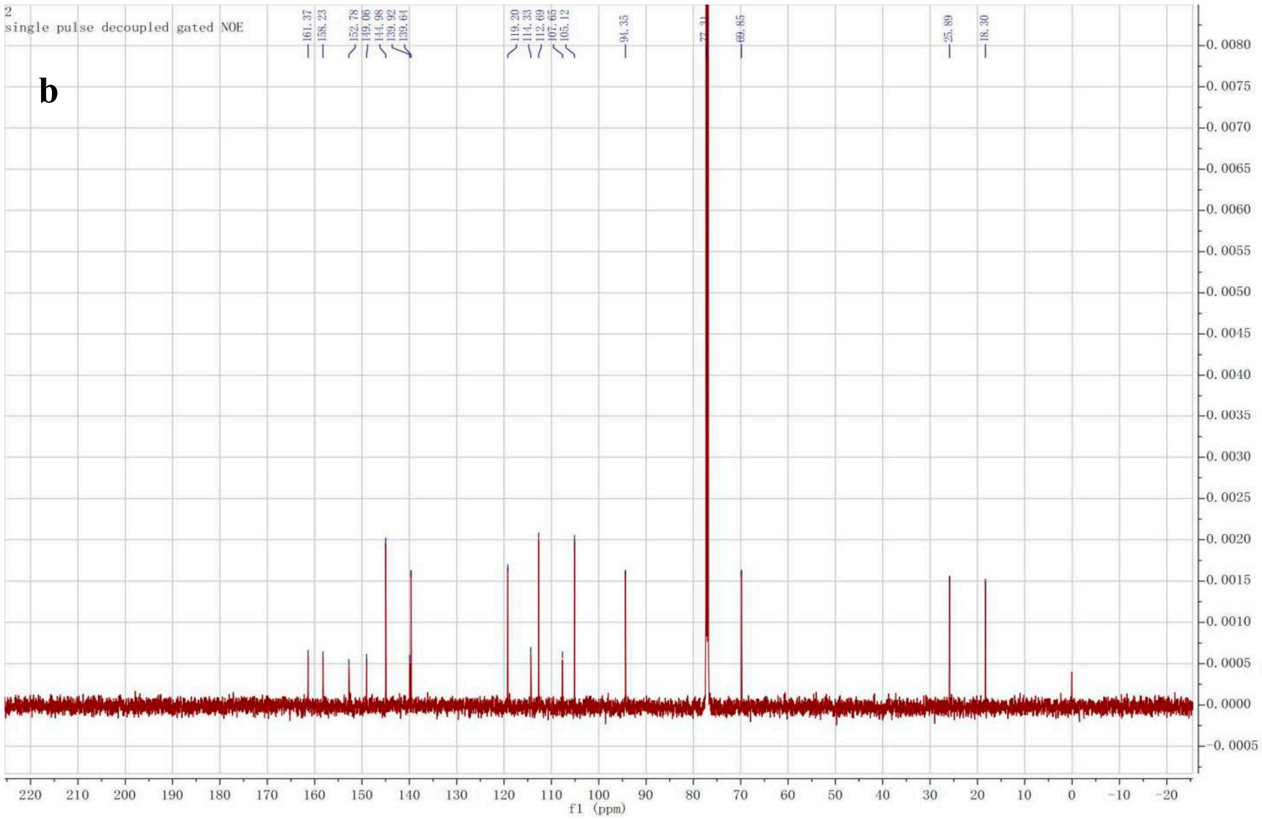

Figure S6 ^{1}H NMR (**a**) and ^{13}C NMR (**b**) spectra of the coumarin **6** (isoimperatorin)

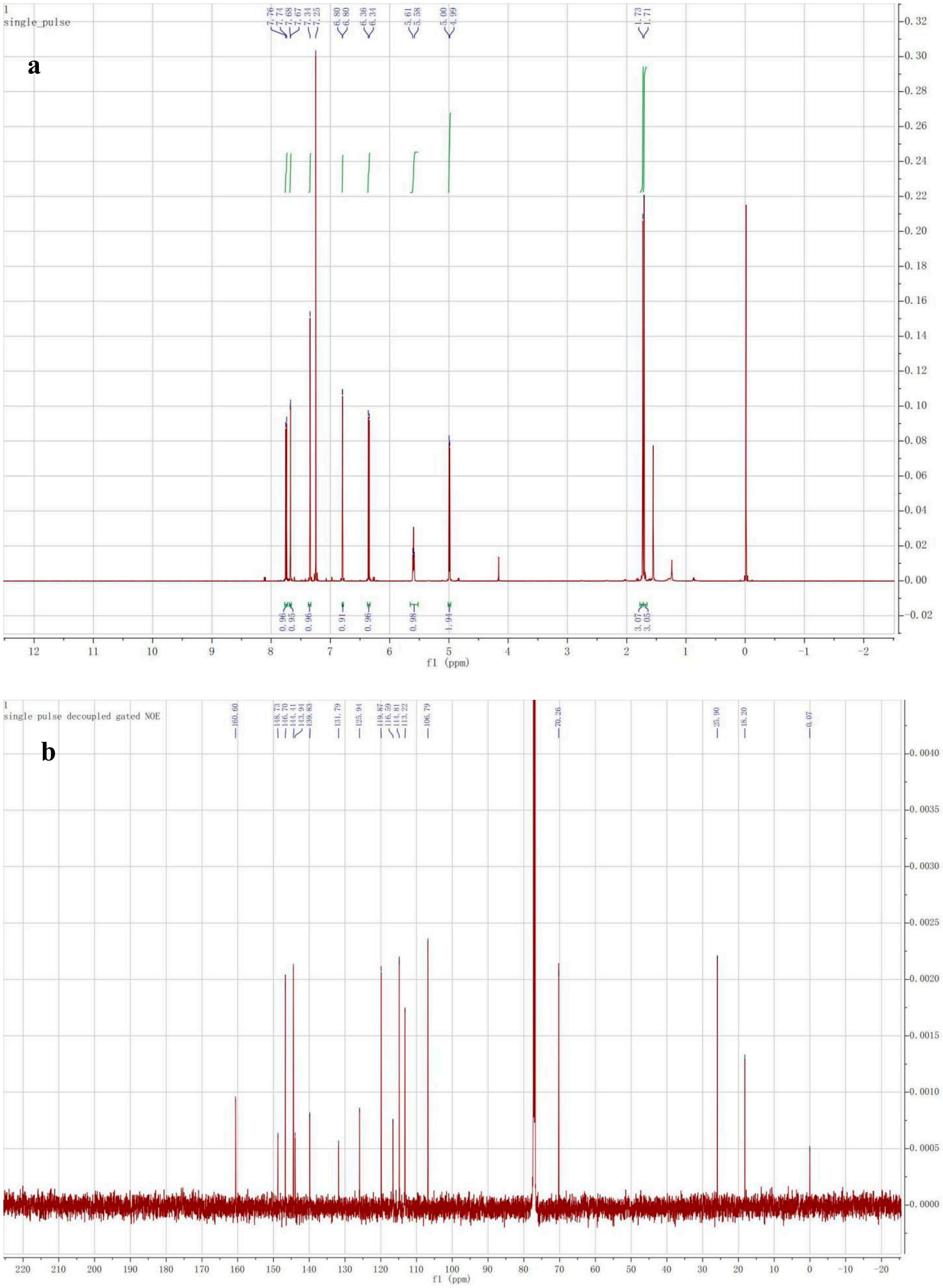

Figure S7 ^{1}H NMR (**a**) and ^{13}C NMR (**b**) spectra of the coumarin **7** (imperatorin)

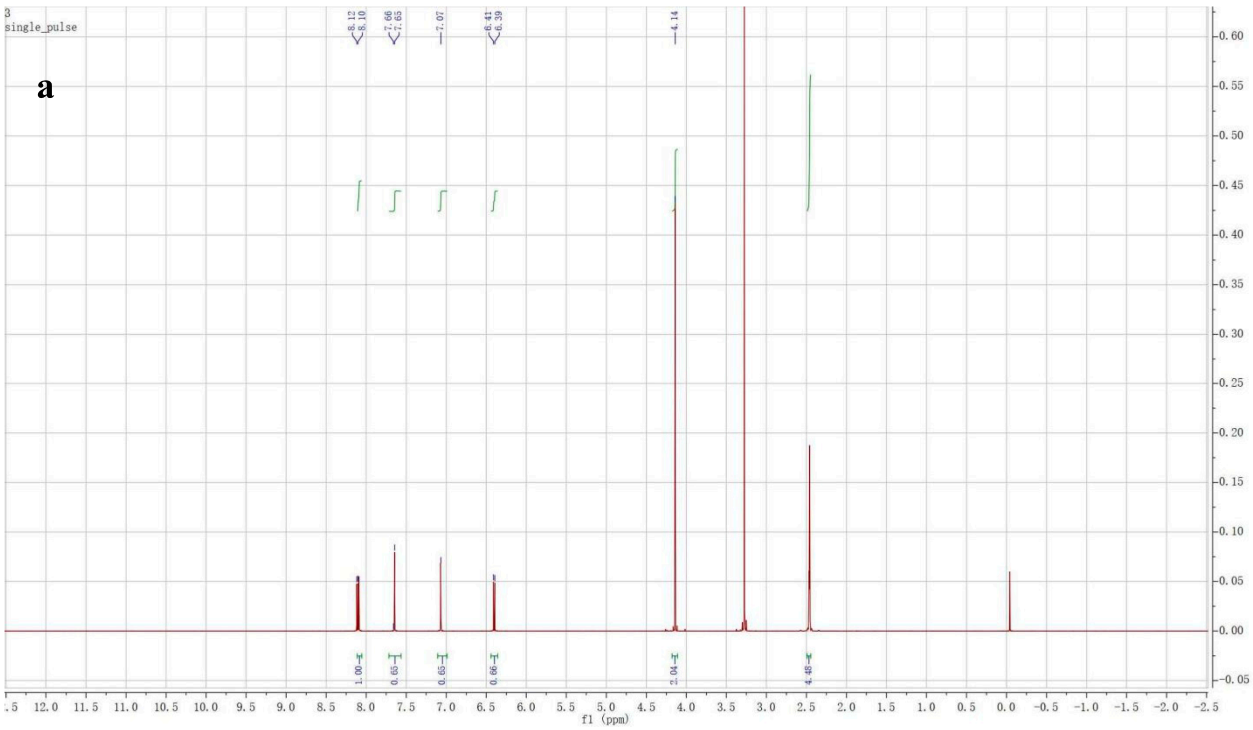

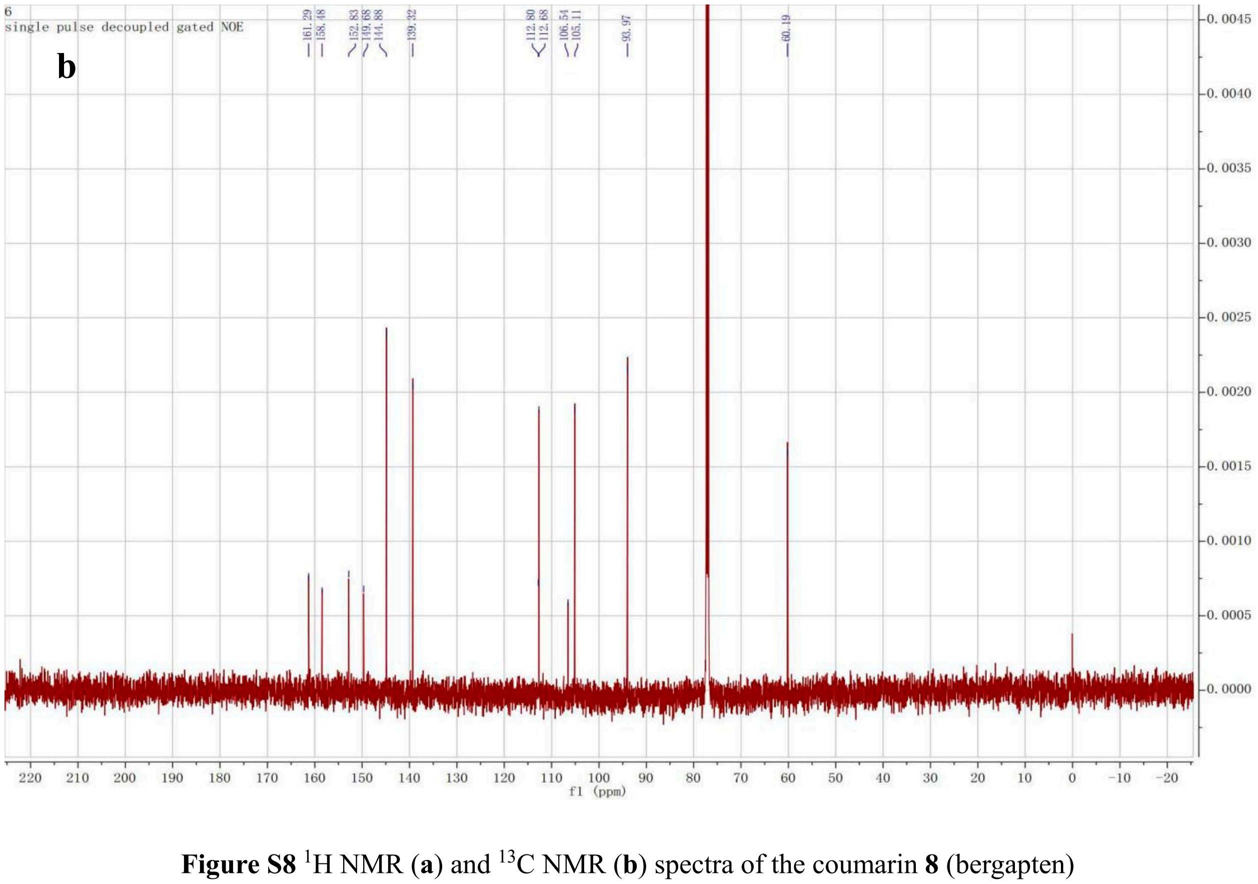

Figure S8 ^{1}H NMR (**a**) and ^{13}C NMR (**b**) spectra of the coumarin **8** (bergapten)
